# Can Quality of Life Tests Be Useful in Patients Affected by Alpha-1 Antitrypsin Deficiency?

**DOI:** 10.3390/jcm13247711

**Published:** 2024-12-17

**Authors:** José María Hernández-Pérez, Hassan Khadour-Khadour, Gema Romero-Romero, Miguel Ángel García-Bello

**Affiliations:** 1 Department of Neumology, Hospital Universitario Nuestra Señora de Candelaria, Carretera del Rosario 145, 38010 Santa Cruz de Tenerife, Spain; ihms4lu@gmail.com (H.K.-K.); gromeroromero97@gmail.com (G.R.-R.); 2 Evaluation Service of the Canarian Health Service, Research Network on Chronicity, Primary Care and Health Promotion (RICAPPS), 38001 Santa Cruz de Tenerife, Spain; miguelgarciabello@gmail.com

**Keywords:** quality of life tests, alpha-1 antitrypsin deficiency, COPD, asthma

## Abstract

Alpha-1 antitrypsin deficiency (AATD) is a genetic condition that predisposes a person to certain diseases over their lifetime, mainly including lung disease (in the form of emphysema) and liver disease (liver cirrhosis). Quality of life questionnaires are instruments designed to quantify the deterioration of a patient’s health. **Background/Objectives**: This study aimed to assess whether certain quality of life tests that are routinely used in clinical practice can be useful for patients with AATD. **Methods**: A sample of AATD patients, with various genotypes, but with the common characteristic that they must have both altered alleles (Pi* ≠ M), participated in the study. Different quality of life tests were used, including the COPD Assessment Test (CAT), COPD and Asthma Sleep Impact Scale, the short form of the Short Form Health Survey, and EuroQol 5 dimensions, and were related to differing clinical and functional characteristics. **Results**: The sample was composed of 54 patients, and slightly more than half of the participants were women (57.4%), with a mean age of 51.5 ± 13.7. The main genotypes were Pi*SZ (43.4%) and Pi*ZZ (34%). In patients under 65 years of age (*n* = 47), those who were actively working could walk a greater distance in the walking test, namely, 573 m (511–629), compared to those who were not actively working, namely, 415.5 m (392–469; *p* < 0.001). Active non-workers had a worse CAT (13.6 ± 7.8 vs. 4.6 ± 4.3; *p* < 0.001). In total, 80% of non-working patients had exacerbations, but only 46. 9% of those who were active, although the association did not reach statistical significance (*p* = 0.068). Having a lower score in the physical component of SF-12 was related to suffering from lung disease (46.0 ± 11.4 vs. 38.4 ± 11.1 (*p* = 0.026)). **Conclusions**: Quality of life tests were able to detect differences and relate them to functional factors such as the distance covered in the walking test, being sensitive and specific in this regard.

## 1. Introduction

Alpha-1 antitrypsin deficiency (AATD) is a genetic condition that predisposes a person to certain diseases over their lifetime, mainly including lung disease (in the form of emphysema) and liver disease (liver cirrhosis) [1]. Various aspects have been assessed to analyze the evolution of patients affected by alpha-1 antitrypsin deficiency (AATD), mainly clinical variables based on functional [2] and radiological tests [3]. We also know that quality of life questionnaires are instruments designed to quantify the deterioration of a patient’s health. Various quality of life tests have been validated for respiratory diseases such as bronchial asthma [4] and chronic obstructive pulmonary disease (COPD) [5]. At present, there are no specific or validated quality of life tests available in patients with AATD, and it is not known whether those commonly used in other patients with respiratory pathologies (Short Form Health Survey (SF-36), St. George’s Respiratory Questionnaire (SGRQ), etc.) or even generic quality of life tests may be useful. The objective of the study was to assess whether certain quality of life tests used in other respiratory diseases can be related to functional tests in patients with AATD.

## 2. Materials and Methods

### 2.1. Study Design

This was an observational, cross-sectional, and descriptive study in which a total of 54 patients with AATD of various genotypes participated. The inclusion criteria were patients who attended the pulmonology outpatient clinic, patients who had undergone genotyping of the SERPINA1 gene, with both alleles altered (Pi* ≠ M), and who gave their informed consent to participate in the study. Various quality of life tests were used, including the COPD Assessment Test (CAT) [6], COPD and Asthma Sleep Impact Scale (CASIS) [7], the short form of the Short Form Health Survey (SF-12) [8], and EuroQol 5 dimensions (EQ5D) [9], and they were related to different clinical and functional characteristics.

### 2.2. Statistical Analysis

Qualitative variables are presented in the form of frequencies and percentages. The percentages were calculated for each group (working vs. not working). Quantitative variables are summarized as mean ± standard deviation and as median (25th percentile–75th percentile). When comparing the groups with two levels, the *t*-test was used when there was a certain normality, while Wilcoxon was used when there was a statistically significant departure from normality.

The *t*-test or Wilcoxon test was chosen according to a normality assessment. The EQ5D5L index was calculated according to the country, in this case, Spain (Morton, 2023) [9]. The CASIS score was calculated after inverting item 6 and considering that a person with no problems would have 0 points and another person with the highest problem score in each of the items would have 100 points. The two SF12 scales were calculated by means of an algorithm. Pearson correlation was calculated for the maximum number of subjects available for each pair of variables.

The study was conducted in accordance with the Declaration of Helsinki and was approved by the ethics committee of Complejo Hospitalario Universitario de Canarias (CHUNSC_2020_23) on 23 March 2021. All patients were informed of the objectives of the study and signed an informed consent form.

## 3. Results

### 3.1. Baseline Characteristics

Slightly more than half of the participants in the sample were women (57.4%), with a mean age of 51.5 ± 13.7, with an API of 29.0 ± 19.4, a Charlson index of 2.5 ± 1.8, and a BMI of 26.4 ± 4.6. In total, 90.6% of the subjects had at least secondary education, and 63.5% had lung disease, mainly COPD (84.8%). The main genotypes were Pi*SZ (43.4%) and Pi*ZZ (34%). The rest of the baseline characteristics of the patients are shown in Table 1.

### 3.2. Relationship Between Quality of Life Tests and Characteristics Clinical and Functional

**Figure 1:**
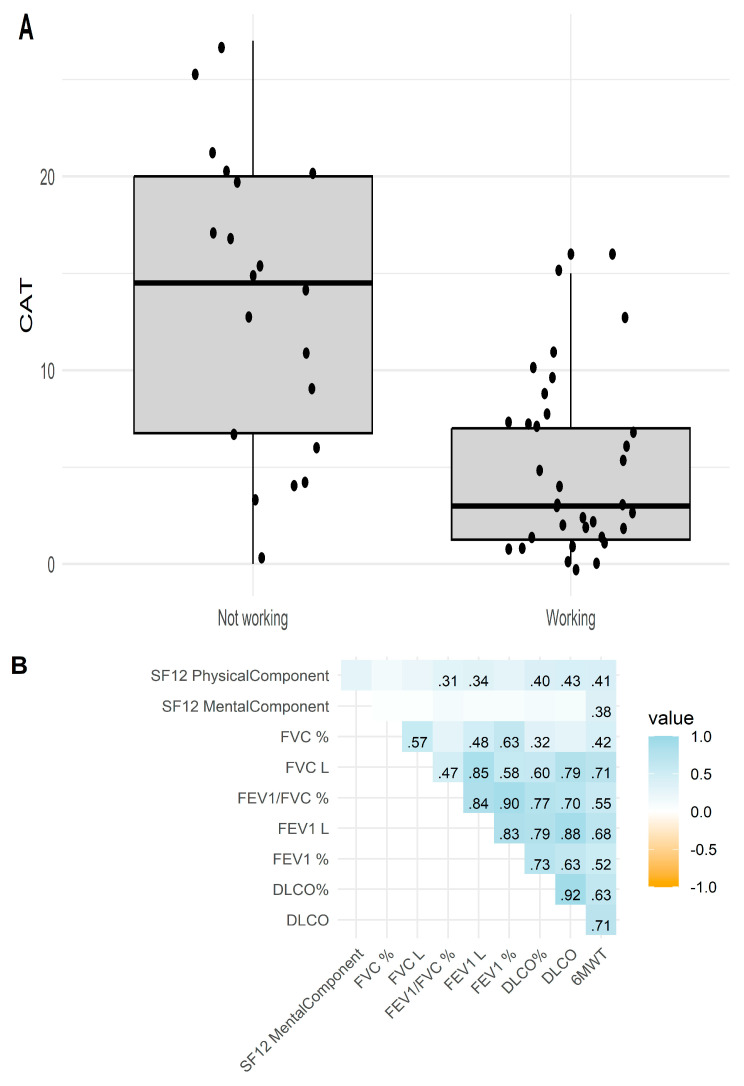

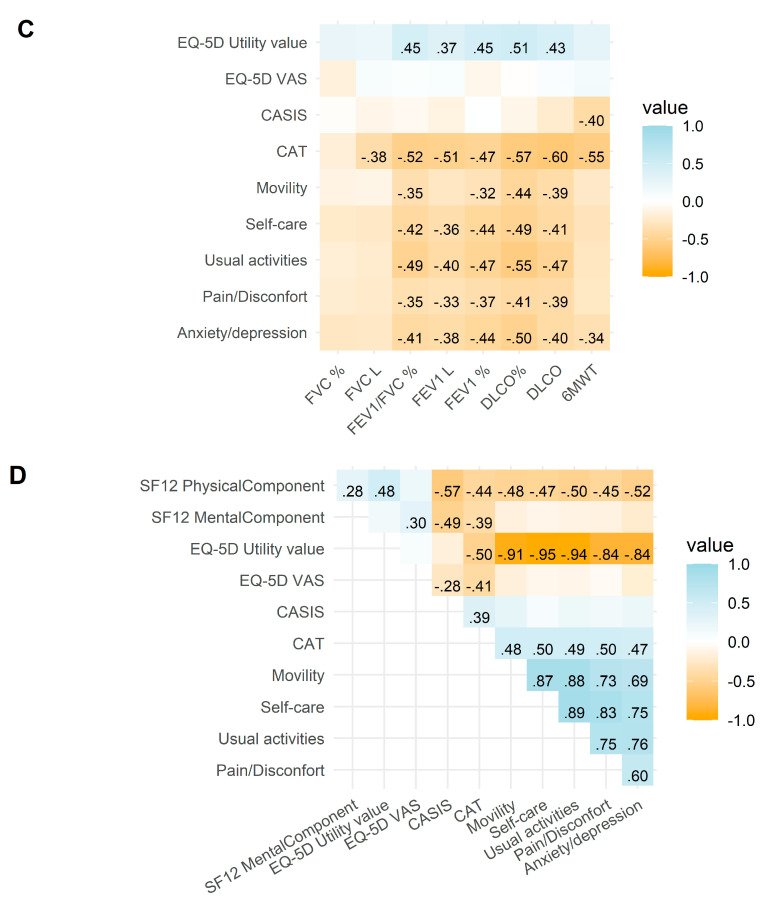
(**A**) Box and whisker diagram representing CAT scores depending on whether the patient was working, in those under 65 years of age. (**B**) Correlation matrix between the two main components of the SF-12 questionnaire and lung function parameters. (**C**) Correlation matrix between quality of life questionnaires and lung function parameters. (**D**) Correlation matrix between the different quality of life questionnaires used. Only significative correlations are shown. FVC: Forced vital capacity. FEV1: Forced expired volume in the first second. DLCO: Diffusion carbon monoxide test. CAT: COPD Assessment Test. CASIS: Asthma Sleep Impact Scale. EQ5D5L: EuroQol 5 Dimensions 5 Levels.

In patients under 65 years of age, those who were actively working reported poorer quality of life scores across the CAT, SF-12 physical component, and EQ-5D-5L index values, as shown in Table 2. No significant differences were observed in pulmonary function variables or AAT levels between working and non-working groups. Active non-workers had a worse CAT (13.6 ± 7.8 vs. 4.6 ± 4.3; *p* < 0.001) (Figure 1A). The CASIS scores were also lower for actively working patients, though this difference was not statistically significant (*p* = 0.082). However, actively working patients achieved a significantly greater distance in the walking test, averaging 573 m (range 511–629), compared to 415.5 m (range 392–469) for non-working patients (*p* < 0.001). Additionally, while 80% of non-working patients experienced exacerbations, this was the case for only 46.9% of actively working patients, although the association did not reach statistical significance (*p* = 0.068), with the exception of the CASIS score.

Overall, the mean SF-12 score for the 54 patients evaluated was 41.6 ± 11.6, significantly below the expected general population mean of 50 (*p* < 0.001; 95% CI = [38.4, 44.8]). Similarly, perceived quality of life as measured by the EQ-5D index was slightly lower than reported values for the general Spanish population, with an average score of 0.7 ± 0.3.

Poorer scores on the SF-12 physical component, CASIS, CAT, and EQ-5D-5L utility value were associated with diminished lung function variables (see Figure 1B,C). Additionally, lower scores on the SF-12 physical component correlated with the presence of lung disease, with values of 46.0 ± 11.4 in unaffected individuals compared to 38.4 ± 11.1 in those with lung disease (*p* = 0.026).

Finally, a higher CASIS score was significantly associated with a shorter walking test distance (r = −0.40; *p* = 0.013). All quality of life scores showed moderate correlations (Figure 1D), with the exception of the CASIS score.

## 4. Discussion

Validated quality of life tests are usually used in routine clinical practice and when clinicians want to assess other aspects of patients’ lives. However, it is not known whether they could be useful in patients with AATD. The present study has shown that quality of life tests help to understand the functioning of patients, offering data and relationships with relevant functional parameters such as distance traveled and DLCO, among others, which alert us about poor prognosis or worse functional and clinical evolution. Sampol et al. [10] already observed that worse values in the CASIS test were related to severity, exacerbations, and worse prognosis in patients with COPD. Choate et al. [11] also used different quality of life tests such as the SGRQ and the Heal-related development of life (HRQoL) in a cohort of patients with AATD, observing worse scores in those subjects who had more exacerbations, a use of chronic home oxygen therapy, and greater degree of dyspnea. Even Ellis et al. [12] were able to detect significant differences in quality of life measured by the SGRQ test when populations using augmentative treatment were compared, although it was not shown to be related to long-term mortality.

In a study comparing populations of patients with AATD with Pi*ZZ vs Pi*SZ genotypes, Torres et al. [13] pointed out that Pi*SZ patients suffered less from lung disease and therefore had a better long-term prognosis than Pi*ZZ patients. However, despite this, the present study observed that when a patient with AATD develops lung disease regardless of the genotype they have (Pi*ZZ, Pi*SZ, rare, or null variants), the quality of life tests were able to detect differences and relate to functional factors such as the distance traveled in the walking test, with these differences being sensitive and specific in this sense.

Celli et al. [14] reported that SGRQ and CAT show that quality of life ratings (QoL) deteriorate with pulmonary function. Current findings support prior research linking COPD patients’ lung function to QoL. The rise in SGRQ and CAT scores reflects physical, psychological, and social effects of COPD on subjects.

Shorofsky et al. [15] also observed that higher test scores on QoL were associated with increased symptom-based exacerbation risk and event-based exacerbation risk. This association occurred mainly in those with undiagnosed COPD. The strongest associations were found with Factor 3 (sleep disturbances and daytime dysfunction). Time to symptom-based exacerbation was shorter in participants with poor sleep quality.

A French study in patients with AATD [16] found a statistically significant association between SGRQ and dyspnea, as well as distance walked in the 6 min walk test. Similarly, another Italian study led by Luisetti [17], with a larger sample size, showed that SGRQ was worse in index cases compared to non-index cases and worse at baseline in patients treated with augmentative therapy compared with untreated patients, but neither study used other quality of life tests to assess other aspects of quality of life, which were used in the present study.

The main limitation of this study is its sample size and cross-sectional and descriptive nature. The temporal sequence of the variables studied could not be established, making it difficult to separate risk factors from prognostic factors; however, the study seems to indicate that quality of life tests may be useful to better define our patients with AATD and recommend their use in our usual clinical practice in this type of patient.

## 5. Conclusions

In conclusion, the results of this study indicate that quality of life tests were able to detect differences and relate to functional factors such as the distance covered in the walking test, with these differences being sensitive and specific in this regard.

## Figures and Tables

**Table 1:** Baseline characteristics of the study patients. BMI: Body mass index. FVC: Forced vital capacity. FEV1: Forced expired volume in the first second. FEV1/FVC: Relation of FEV1/FVC. DLCO: Diffusion carbon monoxide test.

Variable	Mean ± SD *n* (%)	Median ± 25th–75th Percentile
Sex, male	23 (42.6%)	
Age	51.5 ± 13.7	53.5 (45.2–62)
Age at onset of symptoms	35 ± 12.9	32.5 (28–37.8)
Age at diagnosis	43.3 ± 13.9	44 (35–52.8)
Genotypes		
Pi*SZ	23 (43.4%)	
Pi*ZZ	18 (34.0%)	
Pi*SS	5 (9.4%)	
Other genotypes	7 (12.3%)	
Levels of AAT	49.5 ± 25.3	48.2 (29.5–59.6)
Smoking status		
Never smoked	4 (7.4%)	
Ex smoker	26 (48.1%)	
Currently smoking	24 (44.4%)	
Number of packs/year	29 ± 19.4	27 (13–37)
Charlson index	2.5 ± 1.8	3 (1–4)
BMI	26.4 ± 4.6	26.1 (23–30.5)
Cardiac frequency	72.4 ± 4.1	72 (72–73.5)
Respiratory frequency	15.8 ± 1.9	16 (14–16)
SpO2	95.7 ± 2	96 (94–97)
FVC (L)	3.7 ± 1.2	3.6 (2.6–4.6)
FVC (%)	96.9 ± 17.2	98 (90.3–108.4)
FEV1 (L)	2.4 ± 1.3	2.3 (1.2–3.4)
FEV1 (%)	76.2 ± 30.7	83 (41–103)
FEV/FVC %	0.6 ± 0.2	0.7 (0.4–0.8)
DLCO	6.6 ± 3.4	6.7 (4.1–8.8)
DLCO (%)	72.9 ± 26.7	75.2 (53.5–93)
KCO	1.8 ± 2.7	1.3 (0.9–1.6)
KCO (%)	76.4 ± 25.9	76.8 (56–96.5)
RV (L)	2.6 ± 1	2.6 (1.9–3.1)
RV (%)	136.1 ± 48.3	134 (102–161.9)
TLC (L)	6.4 ± 1.3	5.9 (5.3–7.4)
TLC (%)	110.6 ± 24.2	113.2 (102.8–120.6)
Distance traveled	493.8 ± 170.8	524 (415.5–602.5)

**Table 2:** Characteristics of patients working versus not working. The analysis was only performed in under 66 years old. FVC: Forced vital capacity. FEV1: Forced expired volume in the first second. FEV1/FVC: Relation of FEV1/FVC. DLCO: Diffusion carbon monoxide test. CAT: COPD Assessment Test. CASIS: Asthma Sleep Impact Scale. EQ5D5L: EuroQol 5 Dimensions 5 Levels. Quantitative variables are summarized as mean ± standard deviation and as median (25th percentile–75th percentile).

Variables	Not Working (*n* = 15)	Working (*n* = 32)	*p*
Age	58 (48.5–62)	48 (40.5–57)	0.069
Sex female *n* (%)	10 (37.0)	17 (63.0%)	0.576
AAT	48.5 (35–61.2)	52.2 (2.3–59.0)	0.584
FEV1/FVC %	0.56 ± 0.25	0.69 ± 0.18	0.069
FEV1%	50 (35.17–104.25)	89 (64.18–103)	0.154
FEV1 (L)	2.13 ± 1.42	2.81 ± 1.19	0.136
FVC%	92.81 ± 18.69	98.23 ± 17.35	0.369
FVC (L)	3.48 ± 1.03	3.94 ± 1.18	0.202
DLCO%	62.2 ± 25.9	79.2 ± 26.9	0.113
Distance traveled (m)	415.5 (392.3–468.8)	573 (511.3–629.3)	<0.001
CAT	13.6 ± 7.75	4.59 ± 4.32	<0.001
Low impact	5 (33.3%)	29 (90.6%)	<0.001
Medium impact	8 (53.3%)	3 (3.4%)	0.002
High impact	2 (13.3%)	0 (0%)	0.097
SF-12 Physical component	35.3 (24.3–40.8)	49.9 (36.5–54.1)	0.001
SF-12 Mental component	48.5 (36.6–55.3)	55.3 (40.4–58.7)	0.197
CASIS	46.4 ± 26.6	32.2 ± 21.4	0.082
EQ5D5L VAS	60 (50–70)	70 (50–90)	0.129
EQ5D5L index value	0.53 (0.13–0.66)	0.89 (0.74–1)	0.002

## Data Availability

Data are contained within the article.

## References

[1] Hernández-Pérez J.M., Ramos-Díaz R., Vaquerizo-Pollino C., Pérez J.A. (2023). Frequency of alleles and genotypes associated with alpha-1 antitrypsin deficiency in clinical and general populations: Revelations about underdiagnosis. Pulmonology.

[2] Miravitlles M., Turner A.M., Torres-Duran M., Tanash H., Rodríguez-García C., López-Campos J.L., Chlumsky J., Guimaraes C., Rodriguez-Hermosa J.L., Corsico A. (2022). Clinical and functional characteristics of individuals with alpha-1 antitrypsin deficiency: EARCO international registry. Respir. Res..

[3] Strange C., McElvaney N.G., Vogelmeier C.F., Marin-Galiano M., Buch-Haensel M., Zhang X., Chen Y., Vit O., Wencker M., Chapman K.R. (2023). The effect of exacerbations on lung density in α1-antitrypsin deficiency. ERJ Open Res..

[4] Ferrer M., Villasante C., Alonso J., Sobradillo V., Gabriel R., Vilagut G., Masa J.F., Viejo J.L., Jiménez-Ruiz C.A., Miravitlles M. (2002). Interpretation ofi quality of life scores from the St George’s Respiratory Questionnaire. Eur. Respir. J..

[5] Sanjuas C., Alonso J., Sanchis J., Casan P., Broquetas J.M., Ferrie P.J., Juniper E.F., Anto J.M. (1995). The quality of life questionnaire with asthma patients: The Spanish version of the Asthma Quality of Life Questionnaire. Arch. Bronconeumol..

[6] Jones P.W., Harding G., Berry P., Wiklund I., Chen W.H., Kline Leidy N. (2009). Development and first validation of the COPD Assessment Test. Eur. Respir. J..

[7] Miravilles M., Iriberri M., Barrueco M., Lleonart M., Villarrubia E., Galera J. (2013). Usefulness of the LCOPD, CAFS and CASIS scales in understanding the impact of COPD on patients. Respiration.

[8] Schmidt S., Vilagut G., Garin O., Cunillera O., Tresserras R., Brugulat P., Mompart A., Medina A., Ferrer M., Alonso J. (2012). Normas de referencia para el cuestionario de salud SF-12, versión 2 basadas en población general de Cataluña. Med. Clin..

[9] Hernandez G., Garin O., Pardo Y., Vilagut G., Pont À., Suárez M., Neira M., Rajmil L., Gorostiza I., Ramallo-Fariña Y. (2018). Validity of the EQ-5D-5L and Reference Norms for the Spanish Population. Qual. Life Res..

[10] Sampol J., Miravitlles M., Sáez M., Pallero M., Sampol G., Ferrer J. (2023). Poor sleep quality, COPD severity and survival according to CASIS and Pittsburgh questionnaires. Sci. Rep..

[11] Choate R., Holm K.E., Sandhaus R.A., Mannino D.M., Strange C. (2023). Health-related Quality of Life in Alpha-1 Antitrypsin Deficiency-associated Chronic Obstructive Pulmonary Disease. Am. J. Respir. Crit. Care Med..

[12] Ellis P.R., Holm K.E., Choate R., Mannino D.M., Stockley R.A., Sandhaus R.A., Turner A.M. (2023). Quality of Life and Mortality Outcomes for Augmentation Naïve and Augmented Patients with Severe Alpha-1 Antitrypsin Deficiency. Chronic Obstr. Pulm. Dis..

[13] Torres-Durán M., López-Campos J.L., Rodríguez-Hermosa J.L., Esquinas C., Martínez-González C., Hernández-Pérez J.M., Rodríguez C., Bustamante A., Casas-Maldonado F., Barrecheguren M. (2022). Demographic and clinical characteristics of patients with α1-antitrypsin deficiency genotypes PI*ZZ and PI*SZ in the Spanish registry of EARCO. ERJ Open Res..

[14] Celli B.R., Decramer M., Wedzicha J.A., Wilson K.C., Agustí A., Criner G.J., MacNee W., Make B.J., Rennard S.I. (2015). An official American thoracic society/European respiratory society statement: Research questions in chronic obstructive pulmonary disease. Am. J. Respir. Crit Care Med..

[15] Shorofsky M., Bourbeau J., Kimoff J., Jen R., Malhotra A., Ayas N., Tan W.C., Aaron S.D., Sin D.D., Road J. (2019). Impaired Sleep Quality in COPD Is Associated with Exacerbations: The CanCOLD Cohort Study. Chest.

[16] Gauvain C., Mornex J.F., Pison C., Cuvelier A., Balduyck M., Pujazon M.C., Fournier M., AitIlalne B., Thabut G. (2015). Health-related quality of life in patients with alpha-1 antitrypsin deficiency: The French experience. COPD J. Chronic Obstr. Pulm. Dis..

[17] Luisetti M., Ferrarotti I., Corda L., Ottaviani S., Gatta N., Tinelli C., Bruletti G., Bertella E., Balestroni G., Confalonieri M. (2015). Italian registry of patients with alpha-1 antitrypsin deficiency: General data and quality of life evaluation. COPD J. Chronic Obstr. Pulm. Dis..

